# Motility-Independent Vertical Transmission of Bacteria in Leaf Symbiosis

**DOI:** 10.1128/mbio.01033-22

**Published:** 2022-08-30

**Authors:** Tessa Acar, Sandra Moreau, Olivier Coen, Frédéric De Meyer, Olivier Leroux, Marine Beaumel, Paul Wilkin, Aurélien Carlier

**Affiliations:** a Laboratory of Microbiology, Ghent University, Ghent, Belgium; b LIPME, Université de Toulouse, INRAE, CNRS, Castanet-Tolosan, France; c Ghent University, Department of Biology, Ghent, Belgium; d School of Built Environment and Bioeconomy, Tampere University of Applied Sciences, Tampere, Finland; e Royal Botanical Gardens Kew, Richmond, London, United Kingdom; University of Connecticut

**Keywords:** endophytes, phyllosphere-inhabiting microbes, plant-microbe interactions, symbiosis

## Abstract

Hereditary symbioses have the potential to drive transgenerational effects, yet the mechanisms responsible for transmission of heritable plant symbionts are still poorly understood. The leaf symbiosis between *Dioscorea sansibarensis* and the bacterium *Orrella dioscoreae* offers an appealing model system to study how heritable bacteria are transmitted to the next generation. Here, we demonstrate that inoculation of apical buds with a bacterial suspension is sufficient to colonize newly formed leaves and propagules, and to ensure transmission to the next plant generation. Flagellar motility is not required for movement inside the plant but is important for the colonization of new hosts. Further, tissue-specific regulation of putative symbiotic functions highlights the presence of two distinct subpopulations of bacteria in the leaf gland and at the shoot meristem. We propose that bacteria in the leaf gland dedicate resources to symbiotic functions, while dividing bacteria in the shoot tip ensure successful colonization of meristematic tissue, glands, and propagules. Compartmentalization of intrahost populations together with tissue-specific regulation may serve as a robust mechanism for the maintenance of mutualism in leaf symbiosis.

## INTRODUCTION

Heritable symbioses are common in animals, with many examples in invertebrates. More than half of insect species interact with heritable bacteria, such as aphids (Hemiptera) harboring *Buchnera* bacteria (1, 2). These model systems have provided tremendous insights into the cellular mechanisms underlying heritable symbiont transmission (3–5). In contrast to animal symbioses, most well-described plant-microbe symbioses rely on horizontally transmitted symbionts, such as the interactions involving rhizobia or mycorrhizal fungi (6). Heritable transmission of symbionts has been demonstrated for only a handful of plant taxa, and the mechanisms governing symbiont transmission are still poorly understood (7, 8). However, recent evidence suggests that vertically transmitted symbionts may also account for important transgenerational phenotypes (9–11). In addition, mode of transmission has important implications for the evolution of host-microbe associations. Indeed, while horizontally transmitted symbionts are usually vetted through a combination of partner choice and sanctions and rewards, vertical transmission is thought to be an efficient mechanism to establish successful cooperation through partner fidelity feedback (12, 13).

Various plant species harbor possible vertically transmitted bacterial symbionts (14). In the Primulaceae family, 30 out of 35 species of *Ardisia* display small glands at the leaf margin, colonized by *Burkholderia* bacteria (15). In the Rubiaceae family, nearly 500 species engage in leaf nodule symbiosis, also with *Burkholderia* species (15–20). Although many representatives of *Burkholderia* associate with plants, leaf-nodulating bacterial species are more closely related to the newly described genus *Caballeronia*, which mostly contains strains isolated from soil (21). Leaf nodule bacteria lack the genetic ability to fix nitrogen or metabolize phytohormones. Instead, the symbionts provide secondary metabolites that may protect the host against phytophagous insects or competitors (14, 22, 23). Because of their mutual dependence, the study of the molecular mechanisms underlying the associations between heritable leaf bacteria and their hosts is challenging. For example, aposymbiotic seeds of *Psychotria* sp. And *Ardisia crenata* germinate normally but fail to develop more than a few leaves and do not reach maturity (24). Moreover, genomes of leaf nodule bacteria do not encode known signaling pathways such as Nod factors, type III secreted effectors, or plant hormones (14), and the molecular functions enabling colonization and transmission are unknown.

More than 30 years ago, Miller and Reporter (25) used microscopy techniques to describe the presence of a bacterial symbiont in the leaf acumen of *Dioscoreae sansibarensis* (*D. sansibarensis*) but did not identify the bacterium. *D. sansibarensis* is a yam species that reproduces mainly through aerial bulbils and rarely flowers in the wild (25). Interestingly, symbiont-free plants could reportedly be obtained by surface-sterilization of bulbils, although these aposymbiotic plants readily became colonized by bacteria upon transfer to a nonsterile environment. We recently isolated and described these symbiotic bacteria as *Orrella dioscoreae* (*O. dioscoreae*, *Alcaligenaceae*) (26, 27). Leaves of *D. sansibarensis* are heart-shaped and end with a distal acumen or forerunner-tip which exclusively harbors *O. dioscoreae*. The forerunner tip forms the tip of the leaf away from the stem and is the first leaf structure formed as primordia emerge (28). The family *Alcaligenaceae* belongs to the order *Burkholderiales* and includes species with diverse ecological niches. Although some *Alcaligenaceae* bacteria associate with plants (29, 30), most species are isolated from soil or water environments, and rarely from clinical samples (31). Indeed, aside from *O. dioscoreae*, other representatives of the genus *Orrella* were isolated from marine samples (32). In contrast to symbionts of Rubiaceae and Primulaceae, *O. dioscoreae* can be cultured outside the host plant and is amenable to genetic manipulation (27). *O. dioscoreae* can be isolated from vegetative propagules and recent data indicate that the association with *D. sansibarensis* is ubiquitous throughout the range of the host plant. Low phylogenetic congruence between plant and symbiont genetic markers indicates possible horizontal or host-switching transmission, although laboratory experiments show evidence of vertical transmission (27, 33). Moreover, genomes of *O. dioscoreae* strains do not display any of the hallmarks of genome reductive evolution, a common phenomenon in vertically transmitted leaf symbioses (14). The function of the symbiosis remains unknown, but we have previously identified a set of genes in three putative operons that were highly upregulated in the leaf gland compared to axenic cultures (27). Genes of these *smp1*, *smp2*, and *opk* clusters are related to nonribosomal peptide and polyketide synthesis, respectively, and were upregulated >150-fold in the leaf gland versus culture (27).

In this work, we show that the association between *D. sansibarensis* and *O. dioscoreae* is tissue-specific. Using a newly developed gnotobiotic system, we demonstrate that bacteria are transmitted vertically, with possible horizontal transmission relying on bacterial motility and infection of developing apical buds. Our results provide insights into the transmission of a heritable bacterial symbiont in land plants and some of the molecular mechanisms that shape the evolution of leaf-bacteria symbioses.

## RESULTS

### Anatomy of the *D. sansibarensis* leaf gland and its relationship with the symbiotic bacteria.

**FIG 1:**
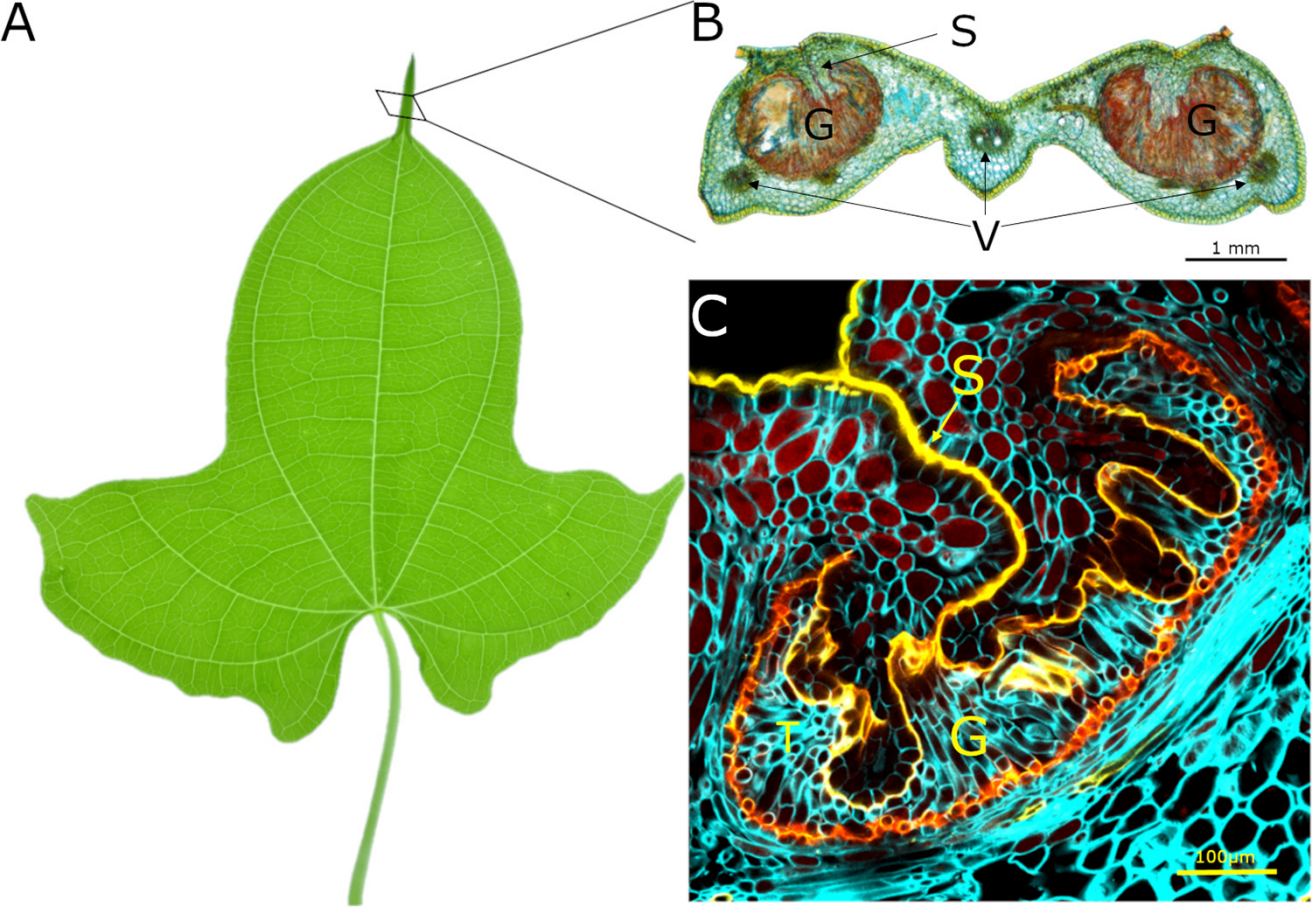
Anatomy of the *Dioscorea sansibarensis* leaf acumen. (A) Juvenile leaf from *D. sansibarensis.* Juvenile leaves are lobed and evolve to heart-shaped leaves in adult plants. Adult leaves can measure up to 46 cm long by 58 cm wide with an acumen at the distal side measuring up to 6 cm. (B) An acumen cross-section. Leaves in all developmental stages contain *O. dioscoreae* in the acumen. In the acumen, two glands (G) that are filled with trichomes (T) and bacteria (stained by acridine orange) residing in mucus can be distinguished. The glands are closed at the adaxial side with a seam (S) running along the long axis of the acumen. Around the glands, several vascular bundles can be found (V). (C) Auramine staining of the acumen shows the thick cuticle (yellow) surrounding the gland (G) that closes up at the adaxial side into a seam (S) and forms a physical barrier to the symbiont (not visible). Plant cell walls are stained with calcofluor white.

**FIG 2:**
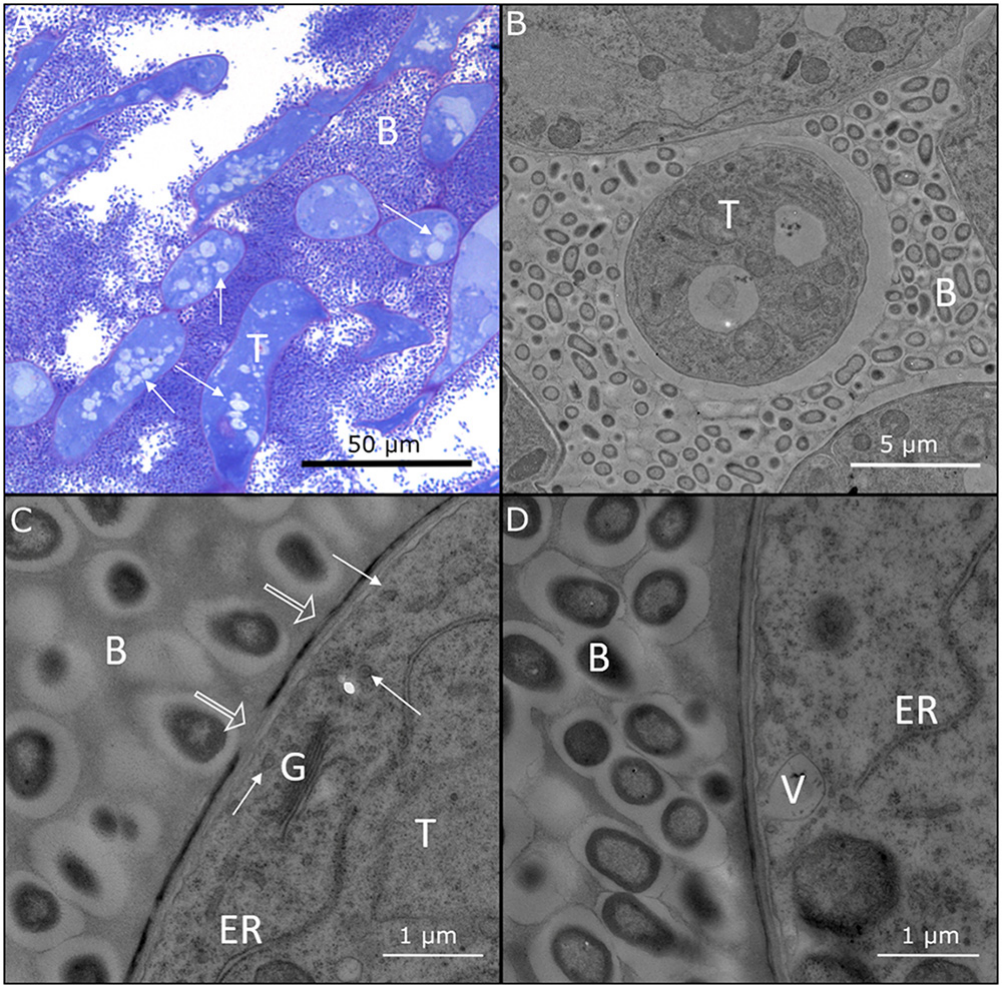
Structure of the trichome-bacteria interface in the symbiotic leaf gland. (A) Light microscopic image of a cross-section of the forerunner tip of *D. sansibarensis.* Trichome cells (T) are densely stained, and multiple lightly colored vesicles are visible (arrows). Around trichomes, many bacteria (B) reside in a mucilage layer. (B) TEM photograph showing the cavity in a symbiotic gland. Trichomes (T) are surrounded by encapsulated bacteria (B) that fill the lumen. (C) TEM image of the interface between the bacteria (B) and a trichome (T) shows the presence of multiple vesicles (white arrows), endoplasmatic reticulum (ER), and Golgi (G). Multiple gaps in the electron dense layer are apparent (hollow arrows). (D) Close-up of the trichome cuticle shows a vesicle (V) merging with the plasmolemma, suggesting transport activity.

To investigate the distribution of the symbiotic bacteria in *D. sansibarensis*, we investigated various tissue for endophytic colonization (Table 1). The acumens on *D. sansibarensis* leaves contain the highest number of viable bacteria, with 2.31 × 10^11^ CFU/g on average compared to <7 × 10^6^ CFU/g for other tissue (Fig. 1, Table 1). Cross-sections of the forerunner tip showed from 2 kidney-shaped glands, and up to 6 glands per acumen in large leaves (Fig. 1B). Glands run along the entire length of the acumen and are lined by a cuticle (25). Glands are closed at the adaxial side, where a remaining suture is apparent (Fig. 1B). A thick cuticle wax layer, which stains intensely with Auramine O, lines the inside the glands. This cuticle layer forms a physical barrier between the mesophyll and the lumen (Fig. 1C). Long vermiform trichomes project into the lumen of the gland (Fig. 1C), which also contains a high density of bacteria (Fig. 1B, Fig. 2A). Trichome cells contain multiple vacuoles and vesicles, indicating intense transport activity to and from the lumen of the gland (Fig. 2A). In addition, bacteria display a thick, electron-lucent capsule with visible membranous projections (Fig. 2B). Trichome cells close to the bacteria-filled lumen contain Golgi, endoplasmatic reticula, and numerous vesicles, some of which were bound to the cytoplasmic membrane (Fig. 2C and D). At this interface between trichome head cells and bacteria, the electron-dense cuticle presents small gaps (Fig. 2C). We did not observe structures resembling bacteria inside plant cells, suggesting a strict extracellular lifestyle. Apical and lateral buds also showed high levels of endophyte colonization. However, leaf lamina and stems contain nearly undetectable quantities of bacteria (Table 1). This suggests that, outside the leaf gland, bacteria only associate with organogenic tissues. We also detected bacteria inside bulbils, the main form of propagule of *D. sansibarensis*. Although difficult to detect by fluorescence microscopy, bacteria may be present in the intercellular spaces of the bulbil growth center, from which new shoots emerge after germination (Table 1, Fig. S2).

**TABLE 1:** Titers of *O. dioscoreae* in various *D. sansibarensis* tissues

Tissue	CFU ± 95% confidence interval	Wt (mg)
Leaf gland	3.20 × 10^10^ ± 3.94 × 10^7^	138.4
Apical bud	6.27 × 10^4^ ± 4.23 × 10^2^	9.3
1 cm^2^ leaf surface	0	26.1
Bulbil growth center	7.83 × 10^4^ ± 6.61 × 10^2^	43.2
Lateral bud	3.53 × 10^4^ ± 1.53 × 10^2^	24.8
Stem under apical bud	4.87 × 10^1^ ± 7.2 × 10^1^	7.65

10.1128/mbio.01033-22.2 FIG S2 Colonization of mCherry-tagged *O. dioscoreae* in the bulbil. Fresh section of growth primordium of *D. sansibarensis* bulbil, colonized by mCherry-tagged *O. dioscoreae*, imaged using confocal microscopy. Gnotobiotic plants were successfully colonized by mCherry-tagged *O. dioscoreae* and grown throughout the life cycle. Bulbils were harvested and after a few months, fresh section were cut through the ‘eyes’ of the bulbil. A. The epidermis (ep) is loose from the underlying cell layers and a primordial plant structure emerges (hollow white arrow) from a pocket ± 1 cm from the surface. B. Red fluorescence (arrows) can be seen surrounding small plant cells, possibly meristematic tissue. Fluorescence seems to be diffused surrounding multiple cells, though concentrated in this one spot underneath the growth initial. Green autofluorescence can be seen. Download FIG S2, PDF file, 0.7 MB. Copyright © 2022 Acar et al. 2022 Acar et al. https://creativecommons.org/licenses/by/4.0/ This content is distributed under the terms of the Creative Commons Attribution 4.0 International license.

### Development of the symbiotic gland.

**FIG 3:**
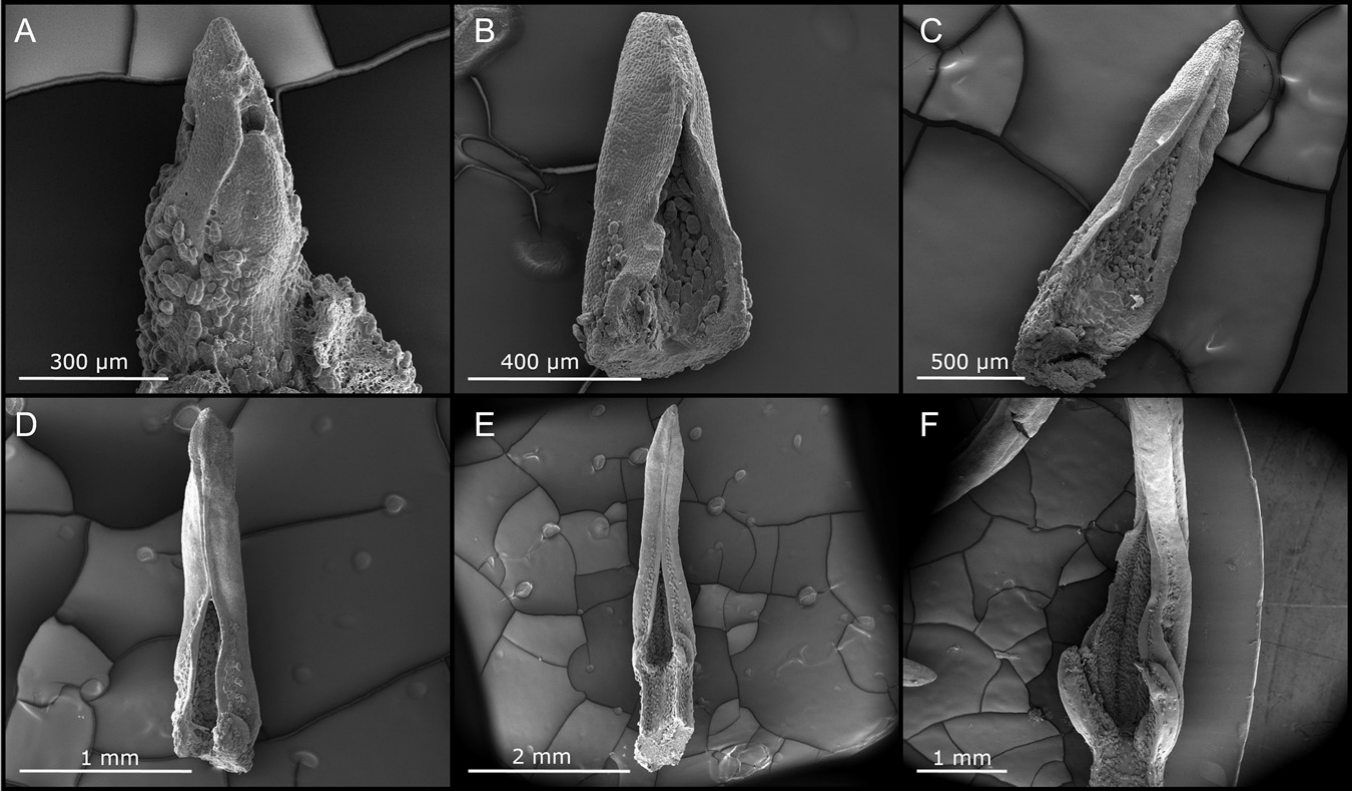
Early development of the *D. sansibarensis* leaf and forerunner tip in the apical bud. (A) Apical meristem surrounded by a leaf primordium. (B) Second youngest leaf of the shoot tip, with many trichomes visible at the adaxial side and an open distal tip. (C) Third youngest leaf of the shoot tip, where the acumen is starting to form, yet remains open. An abundance of trichomes and mucus can be seen at the adaxial surface. (D, E, and F) The third to last, second to last, and last leaf of the shoot tip, respectively. The acumen progressively closes along the long axis and the leaf lamina starts to unfold slowly at the proximal side. Trichomes and mucus are abundant throughout development.

High bacterial loads and evidence for membrane transport activity indicate that the leaf glands are the main site where interaction between the symbiotic partners takes place. To understand how the symbiotic bacteria colonize the newly formed leaf glands, we studied the development of the gland in the apical bud (Fig. 3). The forerunner tip is the first leaf structure formed as primordia emerge. At later stages, the tip of the leaf folds, with margins meeting in the center to form a chamber (Fig. 3B and C). Each apical bud contains 5 to 6 primordial leaves, of which only the three oldest develop a primordial forerunner tip (Fig. 3D–F). The glandular trichomes at the adaxial side of young developing leaves (Fig. S4), in an enclosed space delineated adaxial side of leaves displays high densities of glandular trichomes (Fig. S3). The abaxial side also presents glandular trichomes, albeit in fewer numbers (Fig. S3). Few visible bacteria are embedded in mucus associated with the youngest leaf pair and the apical meristem (Fig. 3A), which is reminiscent of the leaf-enclosed chamber of *Psychotria punctata* (34). We did not observe the same shape of glandular trichomes in the closed acumens. Instead, vermiform glandular trichomes fill the gland together with mucus and the bacterial symbiont. Together, this indicates that bacteria originate from diffuse colonies near shoot meristems and colonize the symbiotic acumens as soon as the structure emerges.

10.1128/mbio.01033-22.3 FIG S3 Adaxial side of an older leaf primordium in the shoot tip, imaged by scanning electron microscopy. Numerous glandular trichomes are present on the adaxial side, and in lesser counts, on the abaxial side of the lamina. Glandular trichomes consist of 1 stalk cell and 5 to 6 glandular cells. Download FIG S3, PDF file, 0.6 MB. Copyright © 2022 Acar et al. 2022 Acar et al. https://creativecommons.org/licenses/by/4.0/ This content is distributed under the terms of the Creative Commons Attribution 4.0 International license.

10.1128/mbio.01033-22.4 FIG S4 Adaxial side of the leaf lamina of a primordial leaf in the shoot tip by scanning electron microscopy. Note the bacteria (arrows) residing in mucus (M) that covers trichomes (T). Download FIG S4, PDF file, 0.5 MB. Copyright © 2022 Acar et al. 2022 Acar et al. https://creativecommons.org/licenses/by/4.0/ This content is distributed under the terms of the Creative Commons Attribution 4.0 International license.

### The shoot tip is a symbiotic hub.

**FIG 4:**
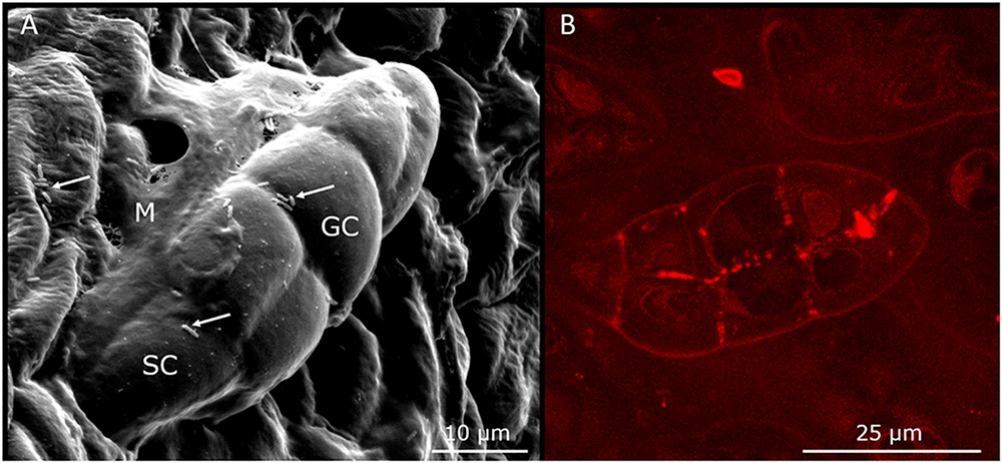
Relationship between *O. dioscoreae* and *D. sansibarensis* in the shoot tip. Scanning electron (left) and confocal (right) microscopy pictures of leaf primordia in the shoot tip show *O. dioscoreae* colonizing glandular trichomes. (A) Trichomes in the apical bud consist of one stalk cell (SC) and 5 or 6 glandular cells (GC). Bacteria (arrow) and mucus (M) surround the trichomes. (B) Confocal microscopy of glandular trichomes in the shoot tip, showing association with mCherry-tagged *O. dioscoreae*. Note the presence of a sticky mucus surrounding the bacteria, especially visible in panel A.

We developed a symbiont-replacement assay to visualize the journey of *O. dioscoreae* from the apical bud to the leaf gland. We first designed a method to generate aposymbiotic plants to facilitate inoculation experiments. Initial attempts to obtain aposymbiotic plants by growing surface-sterilized bulbils in sterile medium as in Miller and Reporter (25) consistently resulted in plants colonized by wild-type *O. dioscoreae* (data not shown). Instead, we obtained aposymbiotic plants by submerging node cuttings in plant growth medium containing a mixture of antibiotics. Aposymbiotic plantlets were then inoculated by depositing cell suspensions of *O. dioscoreae* strains expressing Green Fluorescent Protein (GFP) or mCherry (strains R-71416 and R-71417, respectively) onto the apical bud in otherwise sterile conditions (Fig. S1). Glands of new leaves, which emerged above the point of inoculation, exclusively contained tagged bacteria, while older leaves below did not. This indicates that bacteria colonize symbiotic tissue during early development near the shoot meristem, but do not spread to older tissue via apoplastic or symplastic routes. At the apical bud, bacteria seem to adhere to the trichomes on primordial leaves (Fig. 4).

10.1128/mbio.01033-22.1 FIG S1 Method developed to make aposymbiotic plants and reintroduce a bacterium of interest. A. Node cuttings are taken from adult plants and incubated in a mixture of liquid MS, antibiotics and PPM for 3 weeks. B. After 3 to 4 weeks, a bulbil (b) with its root system become apparent. Multiple leaves have formed from the node and is providing sugars to the plant. C. The bulbil grows its own stem (S) that uses gravitropism to grow up and after the emergence of two leaves, the apical bud becomes visible. D. After confirmation of being aposymbiotic by crushing and plating out the newly developed acumen(s), the plant is reinoculated with a bacterium of interest by dropping 2 μL of the bacterial suspension on the apical bud. Download FIG S1, PDF file, 0.5 MB. Copyright © 2022 Acar et al. 2022 Acar et al. https://creativecommons.org/licenses/by/4.0/ This content is distributed under the terms of the Creative Commons Attribution 4.0 International license.

To investigate if bacteria are transferred to the next generation, we inoculated aposymbiotic plants with mCherry-tagged *O. dioscoreae* R-71417. After 5 weeks of growth in gnotobiotic conditions, we transferred the plants to open pots filled with soil. We harvested bulbils of the plants that survived the transfer to open pots at the end of the growing season and planted the bulbils in soil. Plants germinated from bulbils all contained fluorescent *O. dioscoreae* in their forerunner tips, demonstrating that presence of *O. dioscoreae* at the apical bud is sufficient for the colonization of aerial tissues, including reproductive structures such as bulbils. As a control, we germinated 2 bulbils obtained from aposymbiotic plants, 6 wild-type bulbils, and 4 whole plants harboring GFP-tagged *O. dioscoreae* strain R-71416 in open pots in a growth chamber. Except for the plants inoculated with strain R-71416, none of the leaf glands harvested contained fluorescent bacteria, ruling out possible artifacts due to spontaneous colonization by *O. dioscoreae* that could have lingered in our growth chambers (Table S3). Together, these results demonstrate that the bacterial symbiont is transmitted vertically through the bulbils.

10.1128/mbio.01033-22.8 TABLE S3 Density of bacteria in leaf glands of cocultivated *D. sansibarensis*. Bulbils obtained from plants that were aposymbiotic, wild-type or inoculated with *O. dioscoreae* R-71416 (*egfp, Nal^R^*, *Gm^R^*) were germinated in open pots in a single tray inside a growth chamber. The number of CFU inside leaf glands was determined by serial dilution plating and colony counting on TSA medium containing 30 μg/mL of nalidixic acid (Nal) and 20 μg/mL of gentamycin (Gm) after 48h of incubation as detailed in Materials and Methods. Download Table S3, PDF file, 0.7 MB. Copyright © 2022 Acar et al. 2022 Acar et al. https://creativecommons.org/licenses/by/4.0/ This content is distributed under the terms of the Creative Commons Attribution 4.0 International license.

Although artificial, our symbiont-replacement assay also shows that horizontal acquisition of *O. dioscoreae* is possible. To gain a better understanding of how likely exogenous bacteria are to enter the apical bud, we infected aposymbiotic plants with mixed cell suspensions of GFP-tagged *O. dioscoreae* R-71416 and a wild type *O. dioscoreae* R-71412 in ratios ranging from 1:1 to 1:10^5^, for a total number of approximately 2 × 10^5^ cells per inoculum. Leaf glands were harvested from plants grown in gnotobiotic conditions at 5 weeks postinfection, macerated, and the contents plated on selective medium and nonselective medium to count colonies of tagged and total bacteria, respectively. We detected GFP-tagged bacteria in only 20% of plants inoculated with a dilution factor of 1:100, and none with dilution factors above 1:1000 (Fig. S5A). Importantly, we did not detect significant differences in *in planta* fitness between the two strains (Fig. S5B). This suggests that the number of bacteria establishing in the plant is in the low hundreds.

10.1128/mbio.01033-22.5 FIG S5 (A) Percentage of *D. sansibarensis* plants colonized with GFP-tagged *O. dioscoreae* (strain R-71416) after coinoculation with wild-type *O. dioscoreae* in diminishing ratios from 1:1 to 1:10^5^. (B) Ratios of *O. dioscoreae* strains R-71412 and R-71416 in leaves of plants coinoculated with mixed suspensions of each strain in equal proportions. 4-week old aposymbiotic plantlets were inoculated with mixed cell suspensions of R-71412 (Nal^R^) and R-71416 (Nal^R^, Gm^R^, GFP) and incubated for 4 weeks under sterile conditions. Number of CFU of each strain inside leaf glands were determined by dilution plating and CFU counting on TSA medium containing nalidixic acid or gentamycin (*Resistance to gentamycin*), or by counting colonies on TSA + nalidixic acid expressing GFP versus nonfluorescent colonies (*GFP expression*). The dashed line represents the expected ratio and black horizontal bars indicate the median. The mean observed ratios of the 2 strains are not significantly different from the expected ratio of 1 (“GFP expression” t-test, *P*-value = 0.12; “Resistance to gentamycin” t-test, *P*-value = 0.06). Leaf glands of 4 plants were analyzed for this experiment. Download FIG S5, PDF file, 0.7 MB. Copyright © 2022 Acar et al. 2022 Acar et al. https://creativecommons.org/licenses/by/4.0/ This content is distributed under the terms of the Creative Commons Attribution 4.0 International license.

### Populations of *O. dioscoreae* in the leaf-enclosed chambers and leaf glands are physiologically distinct.

As bulbils and tubers grow from modified shoot buds, we propose that the small colony of *O. dioscoreae* in leaf-enclosed chambers provides the initial inoculum for the developing leaf glands, as well as lateral meristems and the reproductive organs. We hypothesized that bacteria occurring in leaf glands may dedicate their metabolism to symbiotic functions, whereas bacteria in buds may allocate resources for multiplication and transmission. To test if bacterial populations at the leaf glands and at the apical buds have distinct metabolic characteristics, we measured the expression of select *smp* and *opk* genes previously hypothesized to play a central role in this symbiosis (27). Expression levels of *smp* and *opk* transcripts were at least 10-fold lower in apical bud bacteria compared to leaf gland (Table 2). This is likely an underestimation, since transcript levels of target genes were below detection levels in some apical bud samples (Table S4).

**TABLE 2:** Differential regulation of *O. dioscoreae smp* and *opk* gene clusters in leaf gland versus shoot tip by quantitative RT-PCR^*a*^

Replicate	*smp1*	*smp2* ^*b*^	*opk* ^*b*^
Plant 1	58.08	290.01*	35.50*
Plant 2	118.60	296.11*	10.62*
Plant 3	390.72	1478.58*	64.00*
Plant 4	16.33	29.04	10.48*

a Fold changes (2^-ΔΔCt^) in transcript abundance are given in the leaf gland versus the apical shoot tip.

b Values marked with an asterisk (*) are lower-bound estimates, due to a lack of detection in the reference sample (shoot tip, see Table S3).

10.1128/mbio.01033-22.9 TABLE S4 Expression measurement of select genes in shoot tip and acumen by quantitative RT-PCR. *Ct* values of housekeeping gene (gyrB84–85, ODI_R4414) were used to normalise the Ct values of the operons of interest: *smp1* (ODI_R1490), *smp2* (ODI_R1505) and *opk* (ODI_R2249) using primers nrp 88–89, pqqc 90–91 and KASII 82–83, respectively. Ct values >40 indicate lack of detection after 40 PCR cycles. AB: Total RNA isolated from apical bud; LG: total RNA isolated from leaf gland. Total RNA samples were collected during day time from 4 plants grown in soil in growth chamber. Download Table S4, PDF file, 0.7 MB. Copyright © 2022 Acar et al. 2022 Acar et al. https://creativecommons.org/licenses/by/4.0/ This content is distributed under the terms of the Creative Commons Attribution 4.0 International license.

### Motility is dispensable for host colonization, but necessary for horizontal transmission.

**FIG 5:**
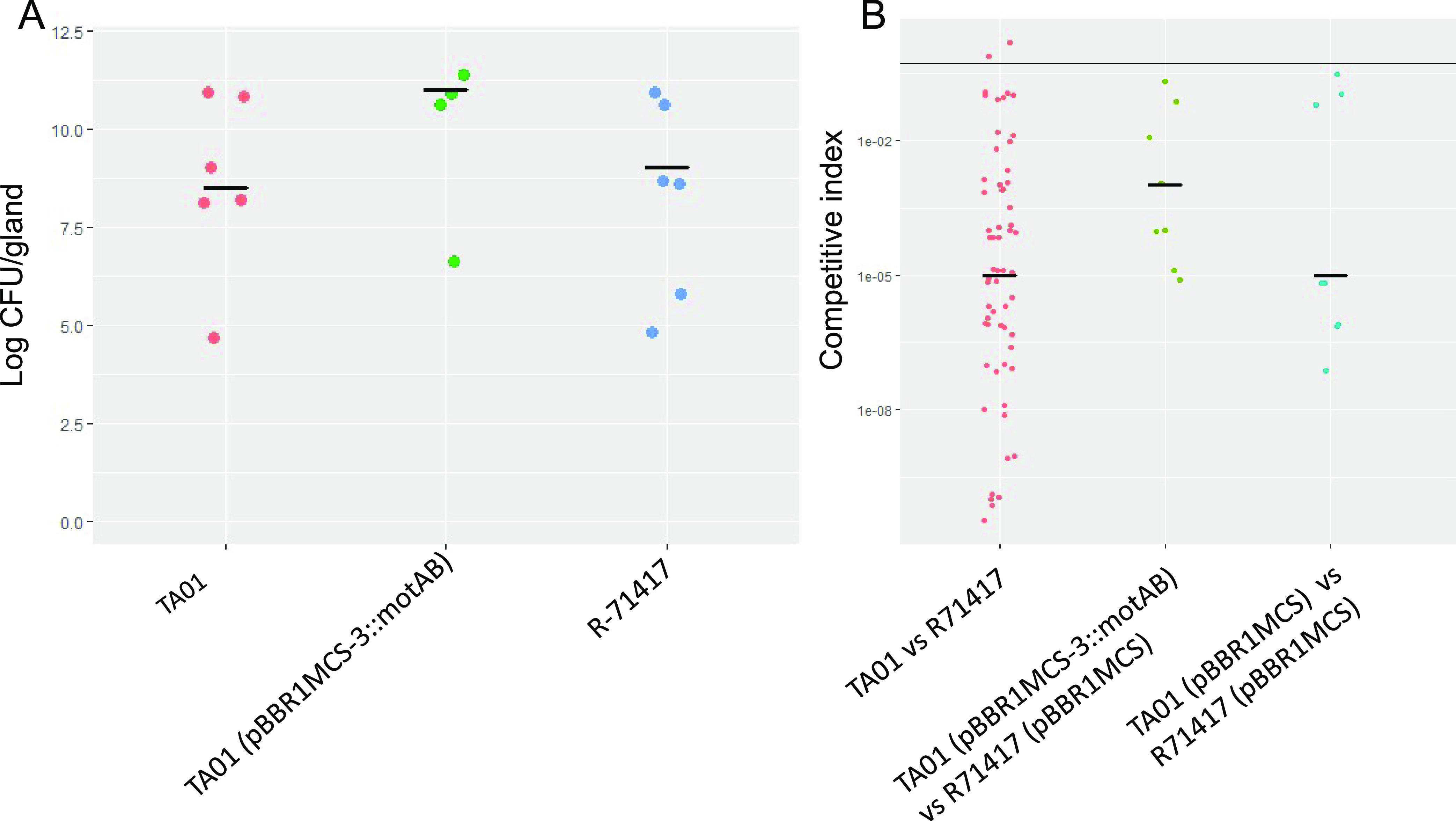
Quantification and competitive index of *O. dioscoreae* strains *in planta*. (A) Titers of *O. dioscoreae* in acumens after single inoculation on the apical bud, one leaf per biological sample. Newly grown acumens were macerated and plated out and growth was quantified by colony counting. Single inoculation with *O. dioscoreae*, either mCherry-tagged strain (R-71417), the motility impaired mutant (TA01), or the complemented strain (TA01 pBBR1MCS-3::*motAB*). There is no significant difference in average bacterial densities between the 3 conditions (pairwise two-sided Student's *t* test, *P > *0.05). (B) Competitive index of *O. dioscoreae* in coinoculations of motility-impaired mutant versus the parental strain. Aposymbiotic plants were coinoculated with a 1:1 mix of motility impaired mutants (strain TA01) and the parental strain (R-71417). As a control, a 1:1 mix of a complemented motility mutant (TA01 pBBR1MCS-3::*motAB*) with the parental strain containing the empty plasmid used for complementation (R-71417 pBBR1MCS) was inoculated into aposymbiotic plants. The expression of a functional copy of *motB in trans* significantly raised the competitive index of strain TA01 *motB^+^* (Two-sided Wilcoxon rank sum test, *P < *0.05). Lastly, to control for the effect of the empty plasmid, strain TA01 pBBR1MCS was coinoculated with strain R-71417 pBBR1MCS (Two-sided Wilcoxon rank sum test, *P > *0.05, compared with the CI of TA01 versus R-71417).

Motility is often required by plant pathogens and symbionts to colonize their hosts (35–39). However, our observation that *O. dioscoreae* do not colonize leaf glands below the inoculation point suggests that movement of bacteria within the plant is limited. Moreover, obligate *Burkholderia* leaf symbionts of Rubiaceae and Primulaceae lack flagella, suggesting that motility is not essential for within-host spread or vertical transmission in leaf symbiosis (15). To test whether flagellar motility is required for colonization of *D. sansibarensis*, we generated motility-impaired strain *O. dioscoreae* TA01 by inserting a kanamycin resistance cassette in a *motB* homolog (locus tag ODI_R2122), which encodes a component of the flagellar motor complex (40). To test the effect of impaired motility *in planta*, we introduced strains R-71417 or TA01 into aposymbiotic plants in gnotobiotic conditions. Colonization rates were high across all conditions, with bacteria in the leaf glands of 59 out of 68 plants (one to four leaves checked per plant). The success rate of inoculations with R-71417, TA01, or TA01 *motB^+^* were similar in single inoculations, with 75%, 66.67%, and 80% of plants successfully colonized, respectively (Chi-squared test, *P*-value > 0.05). Bacterial densities inside leaf glands did not differ significantly between plants inoculated with parental strain R-71417, TA01, or TA01 *motB^+^* (Student's *t* test, *P*-value > 0.05; Fig. 5A). To test whether motility-impaired *O. dioscoreae* strains could still be transmitted vertically, we also transferred some plants inoculated with strain TA01 or R-71417 to open pots in a growth chamber. After 4 months, we obtained 4 bulbils from 3 plants inoculated with TA01, and 2 bulbils from 1 plant inoculated with strain R-71417 (bulbils that formed below the point of inoculation were discarded). The phenotypes of bacteria isolated from bulbils were always consistent with the bacterial genotypes inside the parental plant (Table S5). These results indicate that transmission of motility-impaired *O. dioscoreae* TA01 to bulbils is possible.

10.1128/mbio.01033-22.10 TABLE S5 Isolation of *O. dioscoreae* from bulbils of *D. sansibarensis* inoculated with strains TA01 or R-71417. Bulbils were collected from plants inoculated with *O. dioscoreae* strain R-71417 (*mCherry*, Nal^R^, Gm^R^) or TA01 (R-71417 derivative, *motB::Km^R^*). Plants were kept in soil in a growth chamber, and bulbils formed above the point of inoculation were collected. The number of CFU inside approximately 100 mg of bulbil tissue was determined by maceration and serial dilution plating and colony counting on TSA medium containing 30 μg/mL of nalidixic acid (Nal) and 20 μg/mL of gentamycin (Gm) or kanamycin 50 μg/mL after 48h of incubation as detailed in Materials and Methods. Download Table S5, PDF file, 0.7 MB. Copyright © 2022 Acar et al. 2022 Acar et al. https://creativecommons.org/licenses/by/4.0/ This content is distributed under the terms of the Creative Commons Attribution 4.0 International license.

The inoculum used in our assays contained a large excess of bacteria and these results may not reveal subtle differences in colonization fitness between the strains. To test whether nonmotile strains are outcompeted by motile strains in our assay, we performed coinoculations of aposymbiotic plants with strain TA01 and R-71417 in 1:1 ratio. Bacterial densities of strain TA01 inside leaf glands were between 10 to 10^10^ times lower than R-71417, with a median competitive index of 1.0 × 10^−5^ (Fig. 5B). Moreover, we performed a complementation experiment by coinoculating strains TA01 *motB^+^* and R-71417 pBBR1MCS. The expression of a functional copy of *motB* in *trans* significantly raised the competitive index of strain TA01 *motB^+^*, bringing it to a median value of 1.0 × 10^−3^ (Two-sided Wilcoxon rank sum test, *P < *0.05). These data thus show that flagellar motility is not strictly necessary for plant colonization or vertical transmission but may facilitate horizontal transmission and host-switching.

## DISCUSSION

The unusual tractability of the *D. sansibarensis*/*O. dioscoreae* symbiosis makes this association a valuable model to study the determinants of vertical transmission of plant microbiota, as well as the molecular mechanisms governing the specificity of association of plants with bacteria at the leaf surface. In this work, we show that *O. dioscoreae* symbiotic bacteria are housed in specialized structures at the tip of the leaves, formed by the folding of the leaf margins. These glands hold high densities of bacteria (up to 10^11^ CFU/g) which are separated from the epidermis by a cuticle layer. The cuticle layer and cell wall of trichomes appear thinner in the area directly in contact with the bacteria, with zones of discontinuity in the electron-dense layer (Fig. 2). The plant cuticle acts as a diffusion barrier for water and hydrophilic compounds (41), and gaps in the cuticle layer may enable the diffusion of water-soluble and ionic solutes (42). The presence of numerous vesicles in trichome head cells also supports the hypothesis that trichomes act as a major interface between the symbiotic partners. Although the leaf gland is the most striking feature of the symbiosis, *O. dioscoreae* inhabits other aerial tissues. The distribution of bacteria within the host is however not random. Somatic tissues like stems or leaf lamina contain very few bacteria but shoot organogenic tissues such as apical and lateral buds, as well as vegetative propagules (bulbils), consistently contained symbiotic bacteria (Table 1). Although the bacterial colonies near the shoot apical meristem are more diffuse, bacteria grow within a structure analogous to the leaf-enclosed chamber, previously described in leaf nodulating *Psychotria* species (18, 34). This leaf-enclosed chamber lacks the striking compartmentalization seen in the leaf gland. *D. sansibarensis* thus seems to tolerate the presence of bacteria in contact with shoot meristematic tissue. This close proximity of bacteria at the shoot tip is surprising, since shoot meristems are often thought to be free of microorganisms (43). Because shoot meristems are the hub of aerial organogenesis, tolerance of bacteria near shoot meristematic tissue may be a key feature of the *D. sansibarensis*/*O. dioscoreae* symbiosis that enables a permanent symbiotic association. Unfortunately, we were not able to establish if sexual reproductive structures also contain symbiotic bacteria because *D. sansibarensis* rarely flowers in the wild, and never in cultivation (44).

Inoculation of fluorescent-tagged *O. dioscoreae* at the shoot tip resulted in plants that contained bacteria in all leaves formed above the point of inoculation. Strikingly, we never found evidence of bacteria in the glands of leaves which had emerged prior to the time of inoculation. Microscopic observation also indicates that *O. dioscoreae* attaches to the trichomes of new leaves as they emerge from primordia before the folding of the tip takes place (Fig. 4). Together, this supports the view that growth and distribution of the symbiont is concomitant with leaf development and supported by elongation after gland formation. The fact that we were able to inoculate aposymbiotic plants artificially also suggests that access to the leaf-enclosed chamber remains open after germination. However, we show that out of 2 × 10^5^ *O. dioscoreae* cells, only a few hundred successfully establish in the host after inoculation. This is indicative of stringent barriers to inoculation, similar to some symbiotic systems with horizontal transmission, for example between the bean bug *Riptortus pedestris* and *Burkholderia* (45, 46), or between *Vibrio* and the bobtail squid (47). Together with the fact that all our attempts to force ingress of exogenous bacteria in already symbiotic plants failed (data not shown), we conclude that horizontal symbiont transmission is probably a rare event, in accordance with our previous phylogenetic analyses (33). Whether the potential infection barriers in *D. sansibarensis* are selective to *O. dioscoreae* or if they allow ingress of other bacteria remains to be tested.

Like symbiotic systems with horizontal transmission, flagellar motility contributes to the colonization of new hosts (48–50). The infectivity of *O. dioscoreae motB* mutants was several orders of magnitude lower than that of reference strains (Fig. 5). This suggests that flagellar motility facilitates crossing of host barriers to reach the leaf-enclosed chamber and propagate within the host. Despite this competitive disadvantage, *O. dioscoreae motB* mutants were still capable of infecting aposymbiotic plants and grew to normal densities *in planta.* Moreover, the genome of *O. dioscoreae* lacks genes for alternative types of motilities, such as twitching motility (26). Interestingly, genes linked to chemotaxis or motility functions are entirely lacking from the genomes of some *Burkholderia* leaf nodule symbionts of Rubiaceae and Primulaceae (15, 51, 52). These data strongly suggest that bacterial motility is not required for within-host colonization and transgenerational transmission, but may instead facilitate horizontal transmission and host switching. Indeed, phylogenetic analysis indicate a strict vertical mode of transmission for *Burkholderia* leaf symbiont species entirely lacking a flagellar apparatus (33, 51, 53). Evidence that motility appears dispensable *in planta* indicates that spread of the symbiotic bacteria from the leaf-enclosed chamber to the leaf glands perhaps relies on attachment to specific host structures within the plant. Similar modes of growth and transmission have been hypothesized for vertically transmitted fungal endophytes of grasses: *Epichloë* hyphae attach to host cells at the shoot apical meristem and elongate simultaneously with leaf tissue, allowing asymptomatic colonization of leaves (54). The number of hyphae remains constant in tissue as leaves mature and may be an adaptation to avoid uncontrolled proliferation and triggering of plant defenses (55). In *D. sansibarensis*, the number of bacteria remains constant in apical and lateral buds, as well as bulbil growth centers with approximately 1 to 7 × 10^6^ CFU/g of tissue (Table 1). Similar mechanisms may underlie the heritability of plant microbiota members, including some seed endophytes. Seed endophytes have potential for vertical transmission, but few studies have rigorously investigated transmission routes, e.g., whether reproductive organs were infected before or after development (56). Although selective colonization of shoot apical meristems or leaf-enclosed chambers is the likely transmission route for all leaf nodule symbioses, systematic investigation of homologous leaf-enclosed chambers and reproductive structures of a broad set of plant species using high-resolution techniques may yet reveal that this transmission route has evolved in more taxa. Furthermore, this mode of vertical transmission via colonization of the apical bud appears to be robust: Of more than 50 wild-collected samples of *D. sansibarensis* obtained during previous studies, all contained *O. dioscoreae* in their leaf glands (27, 33). However, this apparent high efficiency may also be explained by a large fitness advantage given to the association: *D. sansibarensis* plants in association with *O. dioscoreae* may have increased chances of survival in the wild. Imperfect transmission of the symbiont may thus yield bacteria-free bulbils that fail to germinate, or seedlings that are quickly eliminated from the population under natural conditions. A systematic analysis of transmission rates in controlled conditions would be required to accurately measure the transmission efficiency of the *D. sansibarensis* leaf symbiosis.

**FIG 6:**
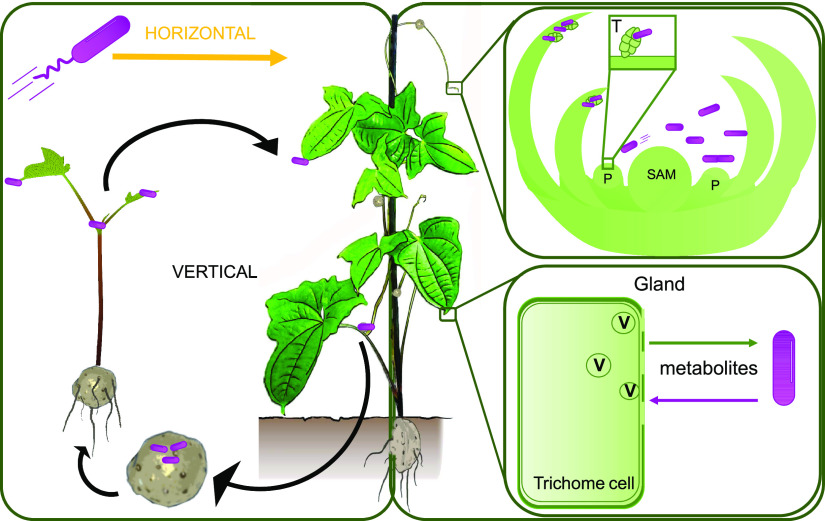
Schematic of *O. dioscoreae* transmission and functional predictions. *D. sansibarensis* harbors symbiotic bacteria (*O. dioscoreae*) which are contained within leaf glands, bulbils, and shoot apical or axillary buds. The apical bud serves as a reservoir for the symbiont to ensure colonization of newly formed aerial organs, such as the forerunner tip, lateral buds, and propagules. Allocation of *O. dioscoreae* cells to bulbils ensures transmission to the next plant generation. Flagellar motility is not essential for vertical transmission but may play a role in occasional horizontal transmission (left panel). The main function of *O. dioscoreae* of this “reproductive” pool residing in the shoot tip may be to serve as an inoculum for the leaf gland and to transmit bacteria to the next generation via bulbils. *O. dioscoreae* attaches to trichomes (T) in the apical bud and grows synchronously with the plant (top right panel). In contrast, the fate of *O. dioscoreae* in the leaf gland may be to provide services to the plant, via exchange of metabolites through the permeable cell wall of specialized trichomes (lower right panel). Leaf gland bacteria are at a reproductive dead-end and are not transmitted to the next plant generation. P, leaf primordium; SAM, shoot apical meristem; V, vesicles; T, trichome.

In addition to controlling bacterial proliferation, specific signals may also control expression of bacterial symbiotic functions in target tissue (57, 58). The *smp* and *opk* genes of *O. dioscoreae* encode putative enzymes of the secondary metabolism, which we hypothesized to play a central role in the leaf symbiosis (27, 33). Genes of the *smp* and *opk* putative biosynthetic gene clusters are highly expressed in the leaf gland, representing nearly 30% of all mRNA (27). However, our data reveal that bacteria in the apical buds express key *smp* and *opk* genes in much lower levels than in the leaf gland (Table 2). This difference in expression may reflect a strategy by the bacteria to maximize use of limited resources in the apical buds toward growth. We propose a model whereby two distinct populations of *O. dioscoreae* are maintained in the plant (Fig. 6): bacteria in organogenic structures (e.g., apical or lateral buds) maintain synchronous growth with host tissue but may not directly fulfill a specific symbiotic function. Bacteria of this “reproductive” pool would have two distinct fates: serve as an inoculum for the leaf gland and transmit bacteria to the next generation via propagules. Bacteria in the leaf gland may provide the main symbiotic services to the plant by expressing a specific gene set but are at a reproductive dead-end. *D. sansibarensis* is an annual plant, with leaves senescing at the end of the season and bacteria presumably dying or at least excluded from the reproductive pool in the plant. It would be interesting to know whether regulation of symbiotic traits depending on localization in the host is important for the evolution of the association, or to protect the host from potentially deleterious metabolites. This will require determining the function of the symbiosis, as well as testing whether bacteria with deregulated symbiotic traits are at a competitive disadvantage for transmission. Investigating whether the plant adapts its immune response to different bacterial genotypes in the leaf gland will also offer key insights into how plants manage beneficial bacteria in aerial tissues.

In conclusion, we provide direct experimental evidence of vertical transmission of symbiotic bacteria in *Dioscorea sansibarensis*. Our work thus provides fundamental insights into the mechanisms governing host colonization and transmission of vertically transmitted bacteria in plants. The unique tractability of the *Dioscorea*/*Orrella* association makes this an appealing model to understand mechanisms of nonpathogenic plant-bacteria interactions in the phyllosphere.

## MATERIALS AND METHODS

### Plant culture and propagation.

Plants were maintained in the greenhouse of the Laboratory of Interactions Plant-Microbe-Environment (LIPME) in Castanet-Tolosan, France. Unless otherwise indicated plants were grown in climate chambers at 28°C, 70% humidity, and a light cycle of 16 h light (210 μmol/m^2^/s), 8 h dark. Chemicals and reagents were purchased from Merck, France unless otherwise indicated. Micropropagation of *Dioscorea sansibarensis* was done using a protocol adapted from Alizedah et al. (59). Node cuttings were collected from greenhouse-grown plants after 2 to 4 months of growth. Explants were surface-sterilized by submerging them in a 5% solution of plant preservative mixture (PPM, Plant Cell Technology, USA) with shaking at 100 rpm for 8 h at 28°C, in the dark. After 8 h, explants were placed in autoclaved growth medium Murashige and Skoog basal salts medium (MS) pH 5.7, supplemented with 2% sucrose, glycine (2 mg/L), myo-inositol (100 mg/L), nicotinic acid (0.5 mg/L), pyridoxine-HCl (0.5 mg/L), thiamine.Cl (0.1 mg/L), and l-cystein (20 mg/L), 200 μg/mL carbenicillin (Meridis, France), 200 μg/mL cefotaxime (Meridis, France), and 0.2% vol/vol PPM. Explants were incubated at 28°C with a 16 h/8 h light cycle for 21 days. The medium was refreshed after 10 days. After 21 days of incubation, explants were transferred to Murashige and Skoog (MS) medium as above, omitting antibiotics. Cuttings were transferred in Magenta GA-7 vessels (Merck), incubated at 28°C with 16h of light until rooting. Effectiveness of the antibiotic treatment to remove *O. dioscoreae* was evaluated by randomly selecting 6 explants per batch, macerating in 0.4% NaCl using a bead mill and plating on Trypticase soy agar (TSA) medium (detailed in “Detection and identification of bacteria”). The lack of visible colonies after 3 days incubation at 28°C was taken as evidence of effective removal of *O. dioscoreae* from entire plantlets.

### Detection and identification of bacteria.

The tip of the leaf was dissected, and the tissue was homogenized using 100 μL 0.4% NaCl and 3 sterile glass beads for 1 min at 30 Hz in a ball mill (Retsch MM 400). One hundred microliters of supernatant were directly plated out on TSA (Sigma) plates and incubated for 2 days at 28°C. If the plate showed growth, one isolate per colony type was picked and identified using colony PCR with primers specific for *O. dioscoreae* (nrdA-01-F, nrdA-02-R; Table S2), or with universal 16S rRNA primers (pA and pH; Table S2) followed by Sanger sequencing. Bacteria were isolated from bulbils as follows: bulbils were washed and surface-sterilized by soaking in 70% ethanol for 10 min, followed by 3 washes with sterile distilled water (dH_2_O). Approximately 100 mg of tissue was dissected from the bulbils with a sterile scalpel under sterile conditions. Tissue was macerated using a sterile plastic pestle in a 1.5-mL microcentrifuge tube containing 500 μL of sterile 0.4% NaCl solution. Serial dilutions of macerates were spotted onto TSA medium supplemented with or without appropriate antibiotics and incubated at 28°C for 48 h. Isolates were typed as described above.

10.1128/mbio.01033-22.7 TABLE S2 Oligonucleotides. Download Table S2, PDF file, 0.4 MB. Copyright © 2022 Acar et al. 2022 Acar et al. https://creativecommons.org/licenses/by/4.0/ This content is distributed under the terms of the Creative Commons Attribution 4.0 International license.

### Inoculation of *D. sansibarensis* with bacteria.

Node cuttings were grown in axenic conditions (25 mL MS + 2% sucrose + 0.2% PPM in Magenta vessel, 28°C, 16 h/8 h light cycle) until a new shoot appeared (after approximately 6 weeks). Aposymbiotic plants were inoculated with a strain of interest as followed: bacterial cultures in the exponential phase of growth were centrifuged (5,000 rpm, 10 min) and washed twice with sterile 0.4% NaCl. Cell suspensions were normalized to OD_600nm_ = 0.2. The biggest leaf at the apical bud was gently pushed aside and 2 μL of the bacterial suspension (OD_600nm_ = 0.2) was gently deposited onto the apical bud (Fig. S1). Plants were transferred to sterile Microbox containers with 50 mL of MS medium, 2% sucrose, and 0.2% PPM at 28°C with a 16 h/8 h light/dark cycle until new leaves emerged. Colonization was evaluated by dissecting a leaf tip and spreading the contents on suitable microbiological medium as described above. For some experiments, plants were transferred to pots with soil and incubated in a growth chamber. Shortly before senescence, plants develop bulbils. These bulbils were harvested and stored in a dark, dry place at room temperature for about 6 months until dormancy broke. Sprouting bulbils were planted in soil and pots were left at 25°C with 16 h of light.

### Bacterial genetics.

The bacterial strains and plasmids used in this section are listed in Table S1. *O. dioscoreae* strain R-71412 is a spontaneous nalidixic acid-resistant strain derived from *O. dioscoreae* LMG 29303^T^ (27). To obtain strain R-71417, a mini-Tn7 cassette containing the *mCherry* reporter gene was introduced into *O. dioscoreae* R-71412 by triparental mating (60). The insertion of the transposon harboring the reporter gene downstream of the *glmS* gene was confirmed by PCR using primers “Mini Tn7 primer forward” and “Mini Tn7 primer reverse” (Table S2). To create a motility-impaired *O. dioscoreae* mutant, a mutant allele of a *motB* homolog (locus tag ODI_R2122) was created by PCR amplification of three overlapping DNA fragments, containing the flanking regions of the gene of interest and the kanamycin resistance cassette from pKD4 (61). The upstream flanking region of the *motB* gene was amplified by using primers motB-UpF-GW and motB-UpR-kan, and the downstream flanking region of the *motB* gene was amplified by using primers motB-DnF-kan and motB-DnR-GW (Table S2). The up- and downstream fragments were fused by SOE-PCR using primers GW-*attB*1 and GW-*attB*2 (Table S2), ligated into pDONRPEX18Tp-SceI-pheS (62) using the Invitrogen BP ligation kit, and transformed by electroporation into E. coli Top10. Suicide plasmids were introduced in *O. dioscoreae* (R-71417) by triparental mating, using E. coli harboring helper plasmid pRK600. Transconjugants were selected on TSA supplemented with kanamycin 50 μg/mL and nalidixic acid 30 μg/mL and incubated for 2 days at 28°C. Counterselection of merodiploid clones was done by spreading on agrobacterium (AB) minimal medium supplemented with 0.2% citrate, 0.1% yeast extract, and 0.1% (wt/vol) *p*-chlorophenylalanine (cPhe) (dl-4-chlorophenylalanine; Sigma-Aldrich). Colonies were screened for loss of trimethoprim resistance on TSA medium supplemented with nalidixic acid 30 μg/mL and kanamycin 30 μg/mL. Selected clones were validated by PCR and whole-genome sequencing to rule out ectopic mutations using Illumina paired-end libraries as described previously. Sequences were deposited in the European Nucleotide Archive with accession number ERR7179810.

10.1128/mbio.01033-22.6 TABLE S1 Bacterial species and plasmids used in this study. Download Table S1, PDF file, 0.7 MB. Copyright © 2022 Acar et al. 2022 Acar et al. https://creativecommons.org/licenses/by/4.0/ This content is distributed under the terms of the Creative Commons Attribution 4.0 International license.

For genetic complementation, the *motAB* locus (locus tags ODI_R2121 and ODI_R2122, including the promoter region) was amplified by PCR using primers motAB-Fwd-KpnI and motAB-rev-SacI (Table S2) and ligated into plasmid pBBR1MCS-3 after restriction with enzymes SacI and KpnI (New England Biolabs). Ligation products were transformed into E. coli Top10 by electroporation. Plasmids were introduced into *O. dioscoreae* by electroporation. Briefly, 1 mL overnight cultures of *O. dioscoreae* were washed 3 times in sterile ultrapure water and resuspended in 40 μL. About 0.5 μg of plasmid DNA were mixed with the cell suspension and transferred to ice-cold 1-mm gap cuvettes (Bio-Rad). Cells were electroporated in a Bio-Rad Gene Pulser Xcell system using settings: 1.8 kV voltage, 25μF, 200 Ω. Transformants were selected on TSA medium supplemented with tetracycline (20 μg/L). We confirmed that MotB is involved in motility in *O. dioscoreae* by measuring the halo of colonies spotted onto soft motility agar (pancreatic digest of casein Bacto peptone [10 g/L], meat extract [3 g/L], NaCl [5 g/L], agar [4 g/L], triphenyltetrazolium chloride [TTC] 0.05g/L) and incubated at 28°C for 48 h. The colony diameter of strain R-71417 (wild type) on motility agar was 6.03 ± 1.11 cm (95% confidence interval [CI]) while strain TA01 (*ΔmotB*) was unable to move beyond the initial spot on the agar (colony diameter of 0.93 ± 0.05 cm; 95% CI). The complemented strain TA01 *motB^+^*, showed intermediate levels of motility with a colony diameter of 2.17 ± 1.23 cm (95% CI). Importantly, we did not notice a difference in growth rates between strains TA01 and R-71417 (data not shown).

### Transmission electron microscopy.

Samples were fixed in 2% (vol/vol) glutaraldehyde (EMS, Hatfield PA, USA) + 0.5% (vol/vol) paraformaldehyde (EMS) in a 50 mM sodium buffer, pH 7.2 at room temperature and under vacuum. After 4 h, the fixative solution was refreshed, and samples were kept at 4°C for 26 days. Samples were rinsed twice in 50 mM cacodylate sodium buffer (pH 7.2) and postfixed in 2% (vol/vol) osmium tetroxide in water for 1.5 h at room temperature in darkness. Samples were dehydrated using a graded ethanol series (10%–100%, 10% increments). Samples were then incubated in propylene oxide (PO) (EMS) twice for 1 h and infiltrated in Epon using a PO/Epon series at 4°C. Samples were embedded in flat embedding molds and polymerized for 48 h at 60°C. Thin sections of 1 μm were cut using a Leica Ultracut E Reichert and contrasted using Uranyless and lead citrate (Delta Microscopies, France). Samples were viewed using a Hitachi HT7700 electron microscope.

### Scanning electron microscopy.

Samples were fixed in 2.5% (vol/vol) glutaraldehyde in 50 mM cacodylate sodium buffer (pH 7.2) for 3 h at room temperature and transferred to 4°C for 2 days. Samples were dehydrated using a graded ethanol series. The samples were dried using a critical point drier (Leica EM CPD 300) using CO_2_ as transitional medium. A platinum coating was applied, and samples were examined using a FEG FEI Quanta 250 electron microscope.

### Light microscopy.

Samples were fixed in 4% (vol/vol) formaldehyde in PEM buffer (100 mM 1,4-piperazinediethanesulfonic acid, 10 mM MgSO_4_, and 10 mM ethylene glycol tetra-acetic acid, pH 6.9) and rinsed in water. Samples were washed in PBS (0.148 g Na_2_HPO_4_, 0.043 g KH_2_PO_4_, 0.72 g NaCl, 0.9 g NaN_3_ in 100 mL dH_2_O, pH 7.1) and dehydrated using a graded ethanol series (30, 50, 70, 85, and 100% [vol/vol]). Samples were polymerized in LR White acrylic resin (medium grade, London Resin Company, UK) using polypropylene capsules at 37°C for 3 days. Semithin sections of 350 nm were cut using Leica UC6 ultramicrotome (Leica Microsystems, Vienna) equipped with a diamond knife. Sections were collected on polylysine-adhesion slides (Carl Roth, Germany) and stained with 1% (wt/vol) toluidine blue O (Merck, Germany) in 1% Na_2_B_4_O_7_ for 20 s at 50°C, rinsed with dH_2_O and mounted in DePeX. Samples stained with calcofluor white and Auramine O were processed as followed: wild-type acumens were fixed in 4% paraformaldehyde in PBS at 4°C overnight, washed twice in PBS, and cleared by subsequently incubating samples in clearing solutions for 1 week at 37°C. The first solution contained 5% vol/vol glycerol, 10% vol/vol sodium deoxycholate, 10% vol/vol urea, 10% vol/vol xylitol, and urea and xylitol concentrations increased to 20% and 30% in week 2 and week 3, respectively. Cleared samples were stained overnight at 4°C in 0.01% calcofluor white and 0.01% Auramine O. Auramine O is a lipophilic fluorescent dye with affinity for regions containing acidic and unsaturated cuticle waxes (63), while calcofluor white stains plant cell walls.

Samples for vibratome sectioning were embedded in 8% agarose, glued upon the cutting stage using superglue (Roticoll 1, Carl Roth, Karlsruhe, Germany) and cut into 30-μm thick sections with a vibrating microtome (HM650V, Thermo Fisher Scientific, Waltham, MA, USA). Sections were stained with 0.5% (wt/vol) astra blue, 0.5% (wt/vol) chrysoidine, and 0.5% (wt/vol) acridine red for 3 min, rinsed in water, dehydrated with isopropyl alcohol, and mounted in Euparal (Carl Roth, Karlsruhe, Germany). All sections were observed using a Nikon Eclipse Ni-U bright field microscope equipped with a Nikon DS-Fi1c camera. To visualize mCherry-tagged *O. dioscoreae* (R-71417) in the shoot tips, fresh plant samples were sectioned with a razor blade and imaged using a laser scanning confocal microscope (Leica TCS SP2). The LAS X software (Leica) was used to process the images.

### Estimation of infection bottleneck.

*O. dioscoreae* strains R-71412 and R-71416 were cultured in Trypticase soy broth (TSB) medium. Cultures in the exponential phase of growth were centrifuged (5,000 rpm, 10 min) and washed twice with 0.4% NaCl. Cell suspensions were normalized to OD_600nm_ = 0.2. Suspensions of R-71416 (GFP-tagged and resistant to gentamicin) were serially diluted with suspensions of the nontagged strain at a constant OD. These suspensions were used to inoculate aposymbiotic plants as described above *(*see Inoculation of *D. sansibarensis* with bacteria). Per condition, 5 plants were inoculated. Plants were left at 28°C with 16 h of light. After 5 weeks, acumens of young leaves were dissected, macerated as described above, and serial dilutions were plated out on selective (TSA medium supplemented with nalidixic acid 30 μg/mL and gentamicin 50 μg/mL), and nonselective (TSA medium supplemented with nalidixic acid 30 μg/mL) medium as described above. To verify that strains R-71412 and R-71416 had similar growth rates *in planta*, leaf glands of plants inoculated with mixed suspensions of each strain in equal proportions were macerated and serial dilutions plated out on selective (TSA, nalidixic acid 30 μg/mL, gentamicin 20 μg/mL) and nonselective media (TSA, nalidixic acid 30 μg/mL). Observed ratios between the two strains were calculated based on the number of CFU on either media, as well as the number of green fluorescent colonies versus nonfluorescent colonies on TSA plus 30 μg/mL nalidixic acid.

### Measurement of gene expression.

Acumen and shoot tip RNA samples were collected during daytime. Apical buds and leaf acumens were ground in liquid nitrogen. RNA samples (four biological replicates per sample) were isolated using the RNeasy plant minikit (Invitrogen) with DNase treatment following the manufacturer’s recommendations. Reverse transcription was performed with 2 μg of total RNA using the reverse transcriptase Superscript II (Invitrogen) and random hexamer primers (Eurofins Genomics, Germany). Quantitative PCRs were conducted in 384-well plates using a LightCycler 480 (Roche) following manufacturer recommendations and the primers shown in Table S2. PCR primers nrp 88–89, pqqc 90–91, and KASII 82–83 correspond to a representative gene from the *smp1* (ODI_R1490), *smp2* (ODI_R1505), and *opk* (ODI_R2249) gene clusters, respectively. The *gyrB* encoding gene was used as an internal standard for sample comparisons. The 2^-ΔΔCt^ method was used for the calculation of relative expression (64).

### Statistical analyses.

All statistical analyses were done using the R software v. 3.6.1 (65) using the built-in functions for statistical tests (t.test and wilcox.test). The package ggplot2 was used for data visualization (66).

### Data availability.

The data sets generated and/or analyzed during the current study are available in the European Nucleotide Archive repository, with the following accession number: ERR7179810.
