# Supplementary material for: Motility-Independent Vertical Transmission of Bacteria in Leaf Symbiosis
Source: mBio. 2022 Aug 30;13(5):e01033-22. doi: 10.1128/mbio.01033-22 (PMC9600174; doi:10.1128/mbio.01033-22)
Supplement: FIG S2 [file mbio.01033-22-s0002.pdf]

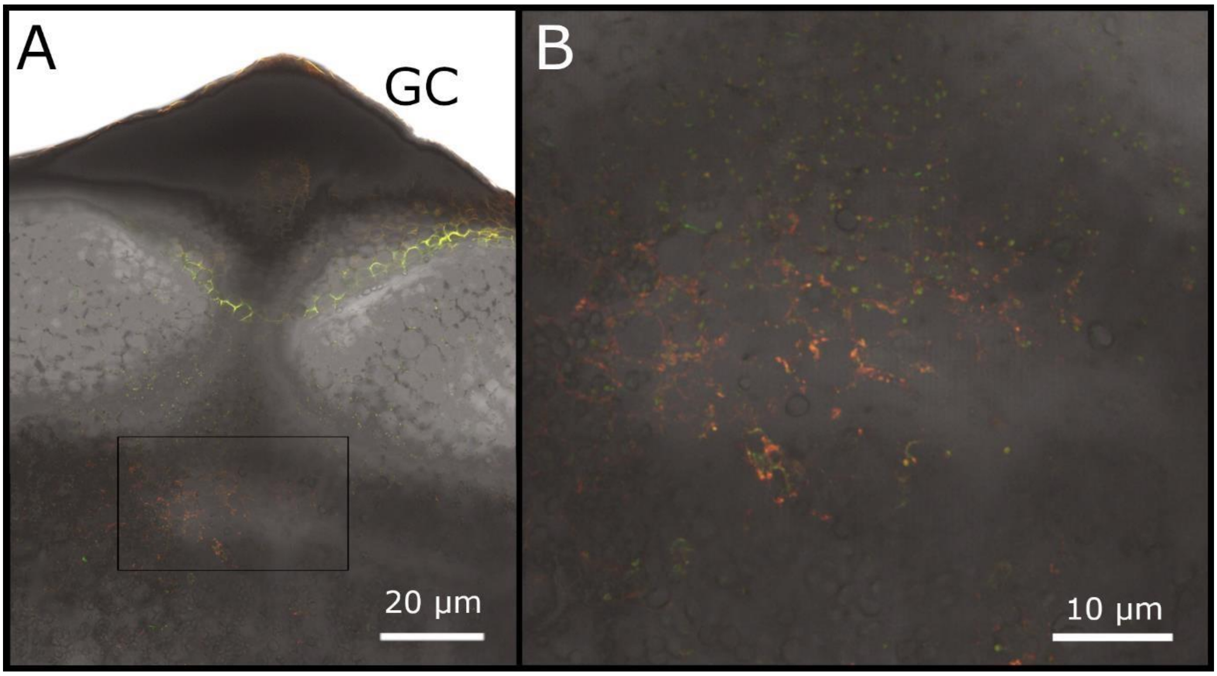

Figure S2: Colonization of mCherry-tagged ***O. dioscoreae*** in the bulbil. Fresh section of growth primordium of *D. sansibarensis* bulbil, colonized by mCherry-tagged *O. dioscoreae*, imaged using confocal microscopy. Gnotobiotic plants were successfully colonized by mCherry-tagged *O. dioscoreae* and grown throughout the life cycle. Bulbils were harvested and after a few months, fresh section were cut through the 'eyes' of the bulbil. (A) The epidermis (ep) is loose from the underlying cell layers and a primordial plant structure emerges (hollow white arrow) from a pocket ± 1 cm from the surface. (B) Red fluorescence (arrows) can be seen surrounding small plant cells, possibly meristematic tissue. Fluorescence seems to be diffused surrounding multiple cells, though concentrated in this one spot underneath the growth initial. Green autofluorescence can be seen.
