# Supplementary material for: Motility-Independent Vertical Transmission of Bacteria in Leaf Symbiosis
Source: mBio. 2022 Aug 30;13(5):e01033-22. doi: 10.1128/mbio.01033-22 (PMC9600174; doi:10.1128/mbio.01033-22)
Supplement: FIG S3 [file mbio.01033-22-s0003.pdf]

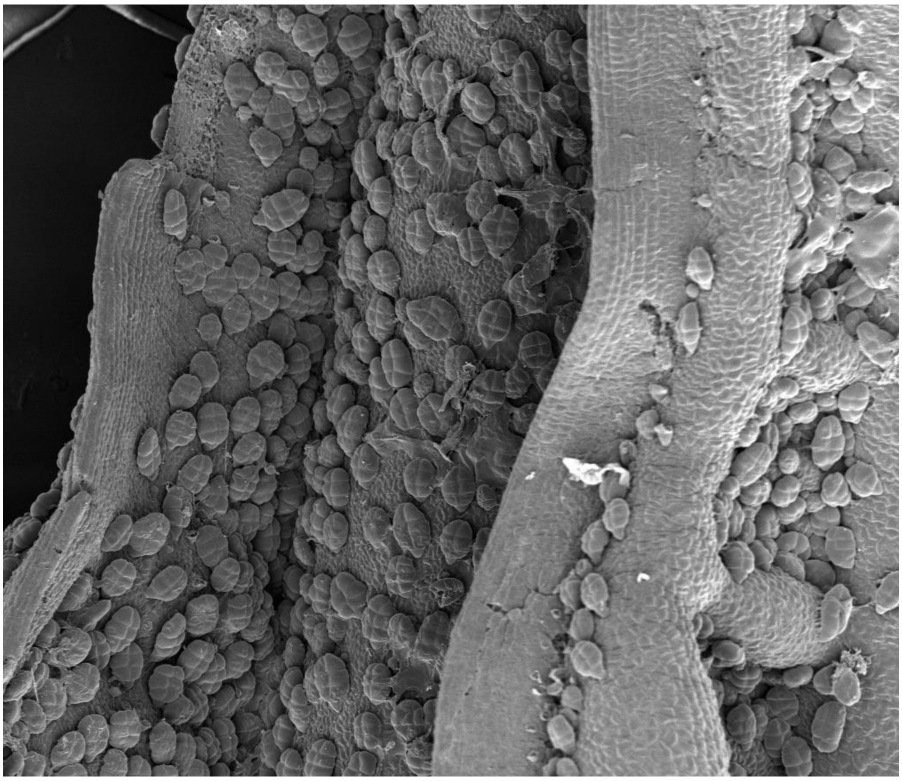

Figure S3: Adaxial side of an older leaf primordium in the shoot tip, imaged by scanning electron microscopy. Numerous glandular trichomes are present on the adaxial side, and in lesser counts, on the abaxial side of the lamina. Glandular trichomes consist of 1 stalk cell and 5-6 glandular cells.
