# Supplementary material for: Motility-Independent Vertical Transmission of Bacteria in Leaf Symbiosis
Source: mBio. 2022 Aug 30;13(5):e01033-22. doi: 10.1128/mbio.01033-22 (PMC9600174; doi:10.1128/mbio.01033-22)
Supplement: FIG S4 [file mbio.01033-22-s0004.pdf]

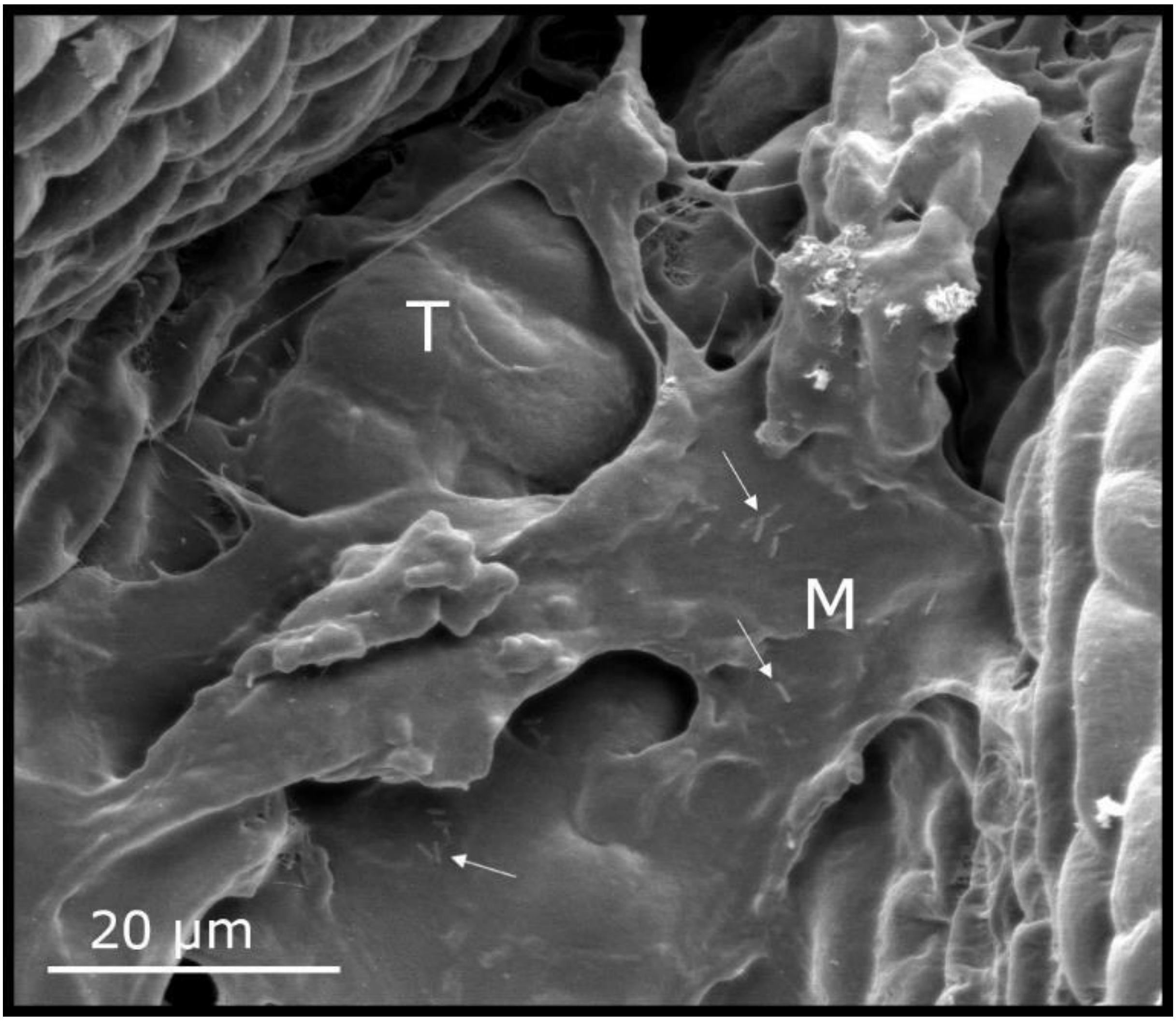

Figure S4: Adaxial side of the leaf lamina of a primordial leaf in the shoot tip by scanning electron microscopy. Note the bacteria (arrows) residing in mucus (M) that covers trichomes (T).
