# Supplementary material for: Motility-Independent Vertical Transmission of Bacteria in Leaf Symbiosis
Source: mBio. 2022 Aug 30;13(5):e01033-22. doi: 10.1128/mbio.01033-22 (PMC9600174; doi:10.1128/mbio.01033-22)
Supplement: FIG S1 [file mbio.01033-22-s0001.pdf]

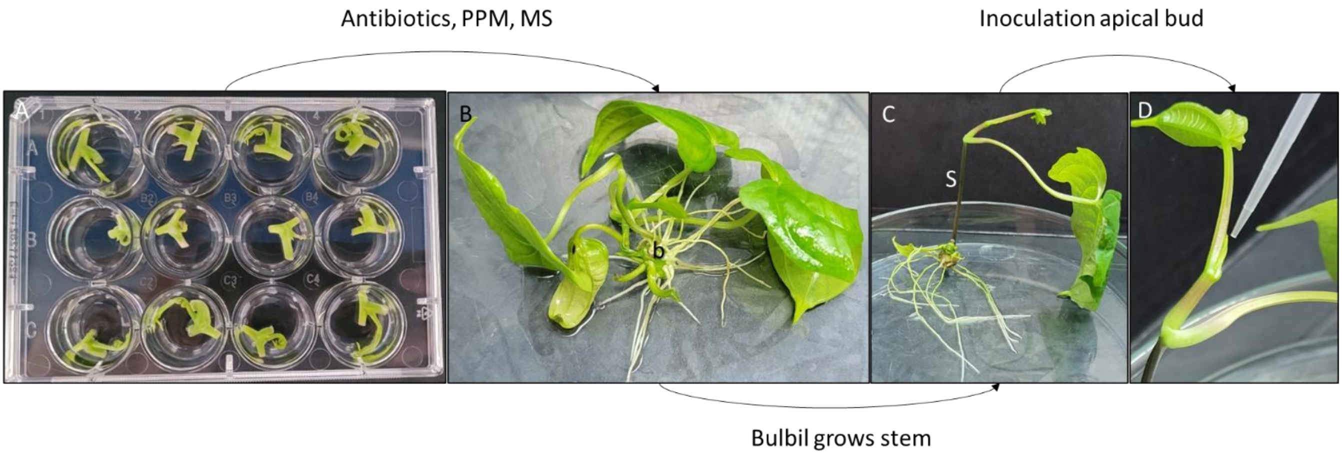

Figure S1: Method developed to make aposymbiotic plants and re-introduce a bacterium of interest
A: Node cuttings are taken from adult plants and incubated in a mixture of liquid MS, antibiotics and PPM for 3 weeks. B: After 3-4 weeks, a bulbil (b) with its root system become apparent. Multiple leaves have formed from the node and is providing sugars to the plant. C: The bulbil grows its own stem (S) that uses gravitropism to grow up and after the emergence of two leaves, the apical bud becomes visible. D: After confirmation of being aposymbiotic by crushing and plating out the newly developed acumen(s), the plant is re-inoculated with a bacterium of interest by dropping 2 µl of the bacterial suspension on the apical bud.
