# Supplementary material for: Motility-Independent Vertical Transmission of Bacteria in Leaf Symbiosis
Source: mBio. 2022 Aug 30;13(5):e01033-22. doi: 10.1128/mbio.01033-22 (PMC9600174; doi:10.1128/mbio.01033-22)
Supplement: TABLE S3 [file mbio.01033-22-s0008.pdf]

Table S3. Density of bacteria in leaf glands of co-cultivated ***D. sansibarensis***. Bulbils obtained from plants that were aposymbiotic, wild-type or inoculated with *O. dioscoreae* R-71416 (*egfp*, *Nal*^{R}, *Gm*^{R}) were germinated in open pots in a single tray inside a growth chamber. The number of CFU inside leaf glands was determined by serial dilution plating and colony counting on TSA medium containing 30 µg/mL of nalidixic acid (Nal) and 20 µg/mL of gentamycin (Gm) after 48h of incubation as detailed in Materials and Methods.

Bulbil type	CFU/gland on TSA	CFU/gland on TSA + Nal + Gm
Aposymbiotic	0	0
	0	0
Wild-type	2×10^7	0
	13×10^7	0
	11×10^7	0
	4×10^6	0
	3×10^7	0
	7×10^7	0
	4×10^8	0
	9×10^4	0
	10×10^7	0
	4×10^8	0
	13×10^7	0
Inoculated with O. dioscoreae R-71416	$>10^6$	$>10^6$
	$>10^6$	$>10^6$
