# Supplementary material for: Motility-Independent Vertical Transmission of Bacteria in Leaf Symbiosis
Source: mBio. 2022 Aug 30;13(5):e01033-22. doi: 10.1128/mbio.01033-22 (PMC9600174; doi:10.1128/mbio.01033-22)
Supplement: FIG S5 [file mbio.01033-22-s0005.pdf]

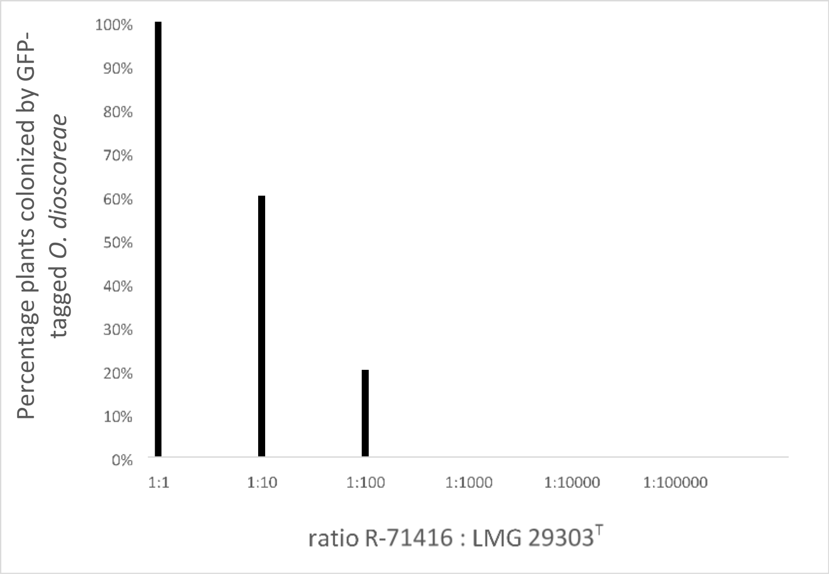

Figure S5A: Percentage of *D. sansibarensis* plants colonized with GFP-tagged *O. dioscoreae* (strain R71416) after co-inoculation with wild-type *O. dioscoreae* in diminishing ratios from 1:1 to 1:10^5.

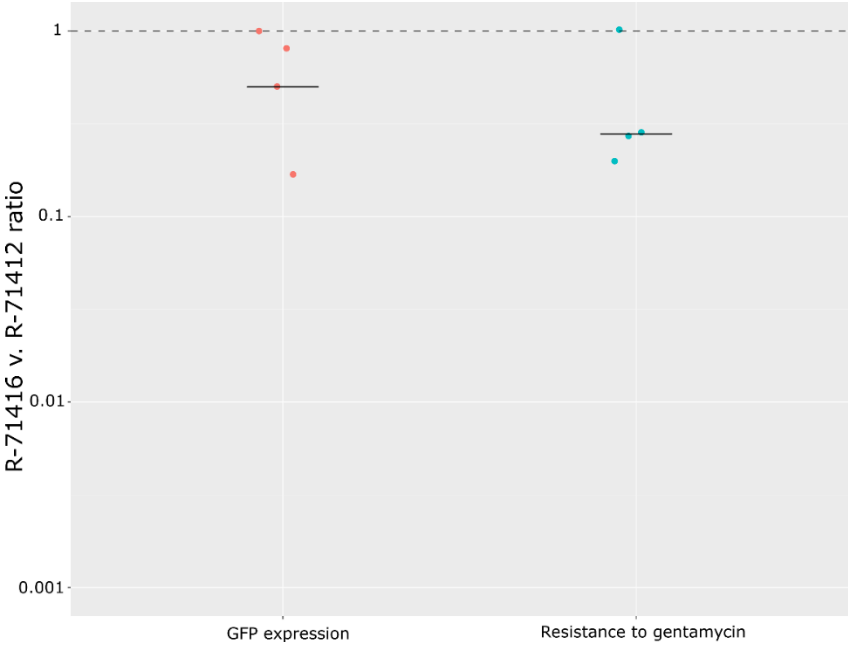

Figure S5B. **Ratios of *O. dioscoreae* strains R-71412 and R-71416 in leaves of plants co-inoculated with mixed suspensions of each strain in equal proportions**. 4-week old aposymbiotic plantlets were inoculated with mixed cell suspensions of R-71412 (Nal^R) and R-71416 (Nal^R, Gm^R, GFP) and incubated for 4 weeks under sterile conditions. Number of cfus of each strain inside leaf glands were determined by dilution plating and cfu counting on TSA medium containing nalidixic acid or gentamycin (*Resistance to gentamycin*), or by counting colonies on TSA + nalidixic acid expressing GFP vs non-fluorescent colonies (*GFP expression*). The dashed line represents the expected ratio and black horizontal bars indicate the median. The mean observed ratios of the 2 strains are not significantly different from the expected ratio of 1 (“GFP expression” t-test *p*-value = 0.12; “Resistance to gentamycin” t-test *p*-value = 0.06). Leaf glands of 4 plants were analyzed for this experiment.
