# Supplementary material for: Motility-Independent Vertical Transmission of Bacteria in Leaf Symbiosis
Source: mBio. 2022 Aug 30;13(5):e01033-22. doi: 10.1128/mbio.01033-22 (PMC9600174; doi:10.1128/mbio.01033-22)
Supplement: TABLE S4 [file mbio.01033-22-s0009.pdf]

Table S4. Expression measurement of select genes in shoot tip and acumen by quantitative RT-PCR. *Ct* values of housekeeping gene (gyrB 84-85, ODI_R4414) were used to normalise the Ct values of the operons of interest: *smp1* (ODI_R1490), *smp2* (ODI_R1505) and *opk* (ODI_R2249) using primers nrp 88-89, pqqc 90-91 and KASII 82-83, respectively. Ct values >40 indicate lack of detection after 40 PCR cycles. AB: Total RNA isolated from apical bud; LG: total RNA isolated from leaf gland. Total RNA samples were collected during day time from 4 plants grown in soil in growth chamber.

***Ct* value**	***gyrB***	***smp1***	***smp2***	***opk***
**AB 1**	33,91	>40	>40	>40
**AB 2**	33,09	>40	>40	>40
**AB 3**	33,12	>40	>40	>40
**AB 4**	34,26	36,85	36,94	>40
**LG 1**	32,55	32,78	30,46	33,49
**LG 2**	33,44	33,46	32,14	36,94
**LG 3**	32,99	31,26	29,34	33,87
**LG 4**	32,77	31,33	30,59	35,12
