# Supplementary material for: Motility-Independent Vertical Transmission of Bacteria in Leaf Symbiosis
Source: mBio. 2022 Aug 30;13(5):e01033-22. doi: 10.1128/mbio.01033-22 (PMC9600174; doi:10.1128/mbio.01033-22)
Supplement: TABLE S5 [file mbio.01033-22-s0010.pdf]

Table S5. Isolation of *O. dioscoreae* from bulbils of *D. sansibarensis* inoculated with strains TA01 or R-71417. Bulbils were collected from plants inoculated with *O. dioscoreae* strain R-71417 (*mCherry*, Nal^R, Gm^R) or TA01 (R-71417 derivative, *motB::Km*^{R}). Plants were kept in soil in a growth chamber, and bulbils formed above the point of inoculation were collected. The number of CFU inside approximately 100 mg of bulbil tissue was determined by maceration and serial dilution plating and colony counting on TSA medium containing 30 µg/mL of nalidixic acid (Nal) and 20 µg/mL of gentamycin (Gm) or kanamycin 50 µg/mL after 48h of incubation as detailed in Materials and Methods.

CFU/g of bulbil	*O. dioscoreae* strain inoculated					
Medium	**R-71417**	**TA01**				
**TSA**	5×10^5	9×10^4	3×10^5	8×10^5	4×10^4	4×10^5
**TSA + Nal30 + Gm20**	2×10^5	9×10^4	1×10^5	4×10^4	3×10^4	2×10^4
**TSA + Nal30 + Km50**	0	4×10^4	1×10^4	9×10^5	1×10^5	3×10^4

Supplementary References

Carlier, A., Cnockaert, M., Fehr, L., Vandamme, P., & Eberl, L. (2017). Draft genome and description of Orrella dioscoreae gen. nov. sp. nov., a new species of Alcaligenaceae isolated from leaf acumens of Dioscorea sansibarensis. *Systematic and Applied Microbiology*, *40*(1), 11–21. https://doi.org/10.1016/j.syapm.2016.10.002

Choi, K. H., Gaynor, J. B., White, K. G., Lopez, C., Bosio, C. M., Karkhoff-Schweizer, R. A. R., & Schweizer, H. P. (2005). A Tn7-based broad-range bacterial cloning and expression system. *Nature Methods*, *2*(6), 443–448. https://doi.org/10.1038/nmeth765

De Meyer, F., Danneels, B., Acar, T., Rasolomampianina, R., Rajaonah, M. T., Jeannoda, V., & Carlier, A. (2019). Adaptations and evolution of a heritable leaf nodule symbiosis between Dioscorea sansibarensis and Orrella dioscoreae. *The ISME Journal*, *13*(7), 1831–1844. https://doi.org/10.1038/s41396-019-0398-8

Fazli, M., Harrison, J. J., Gambino, M., Givskov, M., & Tolker-Nielsen, T. (2015). In-frame and unmarked gene deletions in Burkholderia cenocepacia via an allelic exchange system compatible with gateway technology. *Applied and Environmental Microbiology*, *81*(11), 3623–3630. https://doi.org/10.1128/AEM.03909-14

Kessler, B., de Lorenzo, V., & Timmis, K. N. (1992). A general system to integrate lacZ fusions into the chromosomes of gram-negative eubacteria: regulation of thePm promoter of theTOL plasmid studied with all controlling elements in monocopy. *Molecular and General Genetics MGG*, *233*(1–2), 293–301. https://doi.org/10.1007/BF00587591

Kovach, M. E., Elzer, P. H., Steven Hill, D., Robertson, G. T., Farris, M. A., Roop, R. M., & Peterson, K. M. (1995). Four new derivatives of the broad-host-range cloning vector pBBR1MCS, carrying different antibiotic-resistance cassettes. *Gene*, *166*(1), 175–176. https://doi.org/10.1016/0378-1119(95)00584-1
