# Supplementary material for: Motility-Independent Vertical Transmission of Bacteria in Leaf Symbiosis
Source: mBio. 2022 Aug 30;13(5):e01033-22. doi: 10.1128/mbio.01033-22 (PMC9600174; doi:10.1128/mbio.01033-22)
Supplement: TABLE S2 [file mbio.01033-22-s0007.pdf]

Table S2: Oligonucleotides

Name	Sequence	Tm (°C)	Expected PCR product length (bp)
**pA**	AGAGTTTGATCCTGGCTCAG	57.3	1523
**pH**	AAGGAGGTGATCCAGCCGCA	61.4	
**Mini Tn7 primer fwd**	GCC CTT TCG TCT TCA CCT CG	61.4	1001
**Mini Tn7 primer rev**	AGC TCC TGA AAA TCT CGC CA	57.3	
**GW-attB1**	GGGGACAAGTTTGTACAAAAAAGCAGGCT	65.3	variable
**GW-attB2**	GGGGACCACTTTGTACAAGAAAGCTGGGT	68.1	
**pKD4fwd-2**	TAGGCTGGAGCTGCTTCGAAGTTC	64.4	1473
**pKD4rev-2**	CATATGAATATCCTCCTTAGTTCCTATTCCG	64.2	
**motB-UpF-GW**	TACAAAAAAGCAGGCTCTTCCTGGGCATTCTGCTT	69.5	420
**motB-UpR-kan**	GAACTTCGAAGCAGCTCCAGCCTAAGATCAGCCACAGCAC GAG	60.2	
**motB-DnF-kan**	CGGAATAGGAACTAAGGAGGATATTCATATGATGCGGTG CAGGAATTGAT	71.6	417
**motB-DnR-GW**	TACAAGAAAGCTGGGTCTTCCTGCAAGGTATCTGCATC	61.4	
**motAB-Fwd-KpnI**	GGGGGTACCCATCTGTCGTCCGCCTCC	74.0	2190
**motAB-rev-SacI**	GGGGAGCTCCATCCTCATCGTCACCGAA	71.0	
**nrdA-01-F**	GAACTGGATTCCCGACCTGTTC	53.7	954
**nrdA-02-R**	TTCGATTTGACGTACAAGTTCTGG	56.0	
**gyrB-F**	ACCAGCTTGTCCTTGGTCTG	60.0	60
**gyrB-R**	CGTGCTGTCGGTCAAGGT	60.0	
**nrp-F**	AGGTATAGGGCACGATGAGC	59.0	91
**nrp-R**	CTGGATCTGCGCCACTTC	60.0	
**pqqc-F**	AGGACTTCGGGCTGACACT	59.0	73
**pqqc-R**	TCGAACATGGTGTCGATGAG	60.0	
**KASII-F**	GAGATCGCGGAAAACCAG	59.0	148
**KASII-R**	AAGAACAGGCCATCGACA	59.0	
