# Supplementary material for: Motility-Independent Vertical Transmission of Bacteria in Leaf Symbiosis
Source: mBio. 2022 Aug 30;13(5):e01033-22. doi: 10.1128/mbio.01033-22 (PMC9600174; doi:10.1128/mbio.01033-22)
Supplement: TABLE S1 [file mbio.01033-22-s0006.pdf]

Table S1: Bacterial species and plasmids used in this study

Species	Strain or plasmid	Description	Growth conditions	Reference or source
Orrella dioscoreae	LMG 29303^T		TSA, 28°C	(Carlier et al., 2017)
Orrella dioscoreae	R-71417	Strain R71412 with mini Tn7(Gm)-Ptac::mCherry	TSA + nalidixic acid 30 µg/mL + gentamycin 20 µg/mL, 28°C	This study
Orrella dioscoreae	R-71416	Strain R71412 with mini Tn7(Gm)Ptac-GFP	TSA + nalidixic acid 30 µg/mL + Gentamycin 20 µg/mL, 28°C	This study
Orrella dioscoreae	R-71412	Spontaneous nalidixic acid-resistant mutant of strain LMG 29303^T	TSA + nalidixic acid 30 µg/mL, 28°C	(De Meyer et al., 2019)
Escherichia coli	mini-Tn7(Gm)Ptac ::mCherry		LB broth+ ampicillin 100 µg/ml + gentamycin 10 µg/ml , 37°C	(Choi et al., 2005)
Escherichia coli	mini-Tn7(Gm)PA1/04/03::egfp-a		LB broth+ ampicillin 100 µg/ml + gentamycin 10 µg/ml , 37°C	(Choi et al., 2005)
Orrella dioscoreae	TA01	Derivative of strain R-71417. Kanamycin cassette from pKD4 inserted in *motB* gene	TSA + nalidixic acid 30 µg/mL + gentamycin 20 µg/mL + kanamycin 50 µg/mL, 28°C	This study
Orrella dioscoreae	TA01 *motB*$^+$	Strain TA01 harboring plasmid pBBR1MCS-3::*motAB*	TSA + nalidixic acid 30 µg/mL + gentamycin 20 µg/mL + kanamycin 50 µg/mL, 28°C	This study
Orrella dioscoreae	R-71416	Derivative of strain R-71412 harboring mini-Tn7 transposon from mini-Tn7(Gm)PA1/04/03::egfp-a	TSA + nalidixic acid 30 µg/mL + gentamycin 20 µg/mL, 28°C	This study
Escherichia coli	Top10		LB, 37°C	
Escherichia coli	Top 10 ; pDONRPEX18Tp-SceI-*pheS*		LB + trimethoprim 25µg/ml, 37°C	(Fazli et al., 2015)
Escherichia coli	pRK600		LB + Chloramphenicol 12.5 µg/ml, 37°C	(Kessler et al., 1992)
Escherichia coli	MT102 ; pBBR1MCS-3		LB, 37°C + tetracyclin 10µg/ml	(Kovach et al., 1995)
Escherichia coli	Top10 ; pBBR1MCS-3::*motAB*		LB, 37°C + tetracyclin 10µg/ml	This study
Escherichia coli	SM10 λ-pir ; pUX-BF13		LB + ampicillin 100 µg/mL, 37°C	(Choi et al., 2005)
